# Enriched environment promotes post-stroke angiogenesis through astrocytic interleukin-17A

**DOI:** 10.3389/fnbeh.2023.1053877

**Published:** 2023-02-16

**Authors:** Xiuping Chen, Lingling Liu, Yingjun Zhong, Yang Liu

**Affiliations:** Department of Rehabilitation Medicine, The First Affiliated Hospital of Nanchang University, Nanchang, Jiangxi, China

**Keywords:** enriched environment, astrocyte, IL-17A, angiogenesis, cerebral ischemia/reperfusion

## Abstract

**Objective:**

Our previous studies have revealed that the protective effect of an enriched environment (EE) may be linked with astrocyte proliferation and angiogenesis. However, the relationship between astrocytes and angiogenesis under EE conditions still requires further study. The current research examined the neuroprotective effects of EE on angiogenesis in an astrocytic interleukin-17A (IL-17A)-dependent manner following cerebral ischemia/reperfusion (I/R) injury.

**Methods:**

A rat model of ischemic stroke based on middle cerebral artery occlusion (MCAO) for 120 min followed by reperfusion was established, after which rats were housed in either EE or standard conditions. A set of behavior tests were conducted, including the modified neurological severity scores (mNSS) and the rotarod test. The infarct volume was evaluated by means of 2,3,5-Triphenyl tetrazolium chloride (TTC) staining. To evaluate the levels of angiogenesis, the protein levels of CD34 were examined by means of immunofluorescence and western blotting, while the protein and mRNA levels of IL-17A, vascular endothelial growth factor (VEGF), and the angiogenesis-associated factors interleukin-6 (IL-6), JAK2, and STAT3 were detected by western blotting and real-time quantitative PCR (RT-qPCR).

**Results:**

We found that EE promoted functional recovery, reduced infarct volume, and enhanced angiogenesis compared to rats in standard conditions. IL-17A expression in astrocytes was also increased in EE rats. EE treatment increased the levels of microvascular density (MVD) and promoted the expression of CD34, VEGF, IL-6, JAK2, and STAT3 in the penumbra, while the intracerebroventricular injection of the IL-17A-neutralizing antibody in EE rats attenuated EE-mediated functional recovery and angiogenesis.

**Conclusion:**

Our findings revealed a possible neuroprotective mechanism of astrocytic IL-17A in EE-mediated angiogenesis and functional recovery after I/R injury, which might provide the theoretical basis for EE in clinical practise for stroke patients and open up new ideas for the research on the neural repair mechanism mediated by IL-17A in the recovery phase of stroke.

## Background

Stroke is a major cause of high mortality and permanent disability in adults worldwide because endogenous brain repair cannot fully mitigate the damage it causes (Tsao et al., 2022). After a stroke, the ischemic brain undergoes a series of dynamic alterations, including limited remodeling of the neurovascular unit and dysfunction of the endothelial cells, neurons, and glia (Yang et al., 2021). Among the panel of factors, there is significant evidence suggesting that neurovascular remodeling plays a key role in brain regeneration and functional recovery after stroke, which are closely linked with the outcomes for and the overall survival of patients with stroke (Hatakeyama et al., 2020). This process requires the involvement of astrocytes, vascular endothelial cells, growth factors, and various cytokines, including immune cytokines and chemokines (Schmidt et al., 2013; Lin et al., 2016). Although immune cytokines might have detrimental effects in the acute phase, they exert a beneficial influence on neurovascular remodeling and functional improvement during the recovery phase of stroke (Kleinschnitz et al., 2010; Iadecola and Anrather, 2011).

Interleukin-17A (IL-17A), an immune cytokine belonging to the IL-17 family, is a proinflammatory cytokine mainly produced by activated T cells (Tian et al., 2019). IL-17A may play a biphasic role in ischemic brain injury. At the early stage of stroke, it binds to the IL-17A receptor and acts as a proinflammatory factor that induces a proinflammatory response, which subsequently leads to inherent and adaptive immunity (Kolls and Lindén, 2004). However, IL-17A is mainly produced by astrocytes in the delayed phase and promotes neurogenesis, synaptogenesis, and functional recovery after cerebral ischemia (Lin et al., 2013, 2016). Coupled with neurogenesis, angiogenesis is considered an important factor in functional improvement after stroke. Previous research has shown that astrocytic IL-17A promotes angiogenesis in the ischemic penumbra during stroke recovery, moreover, it was also found that anti-IL-17A monoclonal antibody (mAb) treatment or IL-17A knock-out attenuates its promoting effects on angiogenesis (Zhang et al., 2016).

An enriched environment (EE) is a non-invasive approach comprising voluntary physical activity, social interaction, introduction to new stimuli (by equipping the environment with different toys), and providing a larger space than a standard cage. EE has been shown to play important roles in promoting brain plasticity, angiogenesis, and neurogenesis, ultimately exerting a beneficial influence on brain function and recovery after injury (Kühn et al., 2017; Zhang et al., 2018, 2021). Our previous studies on this subject have proved that exposure to EE following ischemic stroke may lead to astrocyte proliferation and angiogenesis along with trophic factor production, ultimately contributing to functional recovery (Chen X. et al., 2017; Zhang et al., 2017). Moreover, we have revealed that EE promotes angiogenesis by upregulating vascular endothelial growth factor (VEGF) expression (Zhang et al., 2017). In light of the above, we speculate that EE-induced IL-17A expression may participate in the formation of angiogenesis, which may be related to the upregulation of VEGF during stroke recovery.

In this study, we established a rat model of middle cerebral artery occlusion (MCAO) to explore whether the neuroprotective effect of EE on cerebral ischemia/reperfusion (I/R) injury is related to astrocytic IL-17A-mediated angiogenesis, as well as to determine the role of the pro-angiogenesis factors interleukin-6 (IL-6), JAK2, and STAT3 in IL-17A-mediated angiogenesis during the convalescent period of ischemic stroke. This study will provide a basic theoretical basis for designing a comprehensive rehabilitation environment for stroke patients, it will also open up new ideas for the study of the mechanism of IL-17A-mediated nerve repair in the chronic phase of stroke recovery.

## Materials and methods

### Animals, surgery, and housing conditions

Adult male Sprague Dawley (SD) rats [200–220 g, SCXK(E)2020-0018] were purchased from Hubei Experimental Animal Research Center (Wuhan, Hubei, China). All animals were housed in a controlled environment (24 ± 2°C; 12 h light/dark cycle, 50–60% relative humidity) with free access to filtered clean water and standard laboratory food for 3 days to adapt to the surrounding environment prior to initiating experiments. Animals were randomized for treatments that were blinded to personnel carrying out the surgical procedures, injections, and physiological function analysis. No statistical methods were used to predetermine sample sizes in animal studies, but our sample sizes are like those generally employed based on our previous work (Chen and Zhang, 2022). Finally, a total of 60 mice were used in this study. All animal handling and experimental procedures were performed in line with the Care and Use of Laboratory Animals guidelines published by the China National Institute of Health and approved by the Institutional Animal Care and Use Committee of Nanchang University.

Transient MCAO rat models were constructed as previously described in Chen and Zhang (2022). In brief, SD rats were anesthetized with isoflurane through a face mask and all efforts were made to minimize the suffering of the animals. A linear 2 cm skin incision was made in the midline of the anterior neck, and the common carotid artery (CCA), external carotid artery (ECA), and internal carotid artery (ICA) were each exposed in turn. The ECA was ligated at the level of the bifurcation, after which a monofilament nylon filament (Beijing Cinontech Biotech Co., Ltd., Beijing, China) was gently advanced into the ICA through the right CCA to a point about 18 mm distal to the bifurcation; finally, the right MCA was occluded. Sham-operated rats underwent an identical surgery, except that a nylon filament was not inserted. After recovering from anesthesia, the neurological deficit was scored on a five-point scale. Rats with scores of 0 or 4 were excluded from the study (Wu Q. et al., 2020). Only rats with scores of 1–3 were considered suitable for inclusion in subsequent trials.

Twenty-four hours after MCAO, rats in EE groups were housed in a spacious cage (50 × 75 × 90 cm^3^) containing novel objects such as climbing ladders, plastic tunnels, shelters, toys, tubes of different shapes, and running wheels for voluntary exercise (10–12 rats/cage). Rats in the standard environment were housed in a standard cage (20 × 32 × 44 cm^3^) with no objects (3–4 rats/cage).

### Experimental groups and drug administration

**FIGURE 1:**
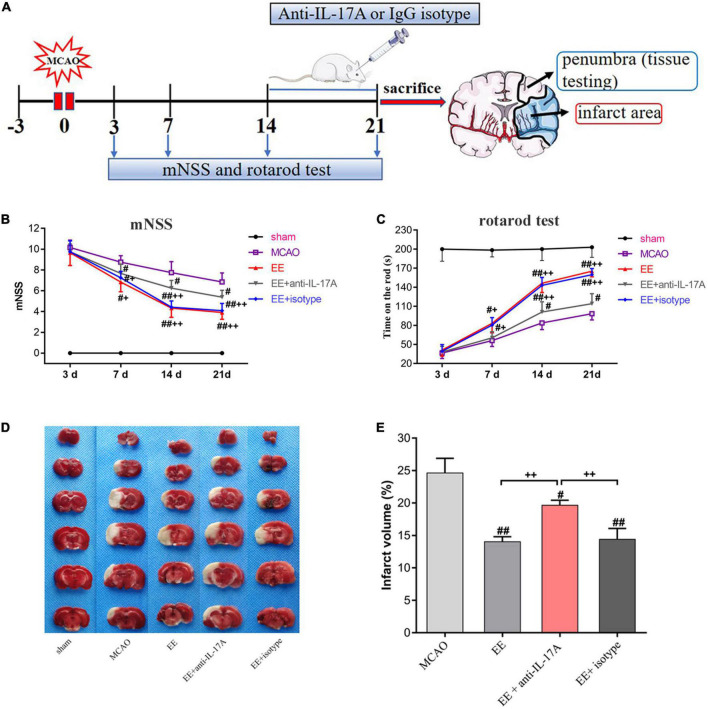
Schematic drawing of the experimental timeline and effect of enriched environment (EE) treatment on functional outcomes and infarct volume 21 days after middle cerebral artery occlusion (MCAO). **(A)** Timeline of behavioral testing and intracerebroventricular injection of interleukin-17A (IL-17A)-neutralizing mAb [2 μg, Intracerebroventricular (i.c.v.)] or IgG1 isotype after stroke. At 21 days after behavioral testing, the rats were sacrificed for tissue testing. **(B)** Evaluation of the neurological function of rats in the sham, MCAO, EE, EE + anti-IL-17A, and EE + isotype groups using modified neurological severity scores (mNSS) scores, *n* = 12 in each group. **(C)** The results of the rotarod test in five groups through measurement of time on the rod(s), *n* = 12 in each group. **(D)** Representative 2 mm coronal sections of each group. **(E)** Quantitative analysis of infarct volume percentage in each group. Sham-operated rats had no ischemic tissue (data not shown). Data are presented as mean ± SD. ^#^*P* < *0.05*, ^##^*P* < *0.01* vs. MCAO group; ^+^*P* < *0.05*, ^++^*P* < *0.01* vs. EE + anti-IL-17A group.

Experimental groups were assigned in a randomized and blinded manner. The rats were divided into five groups: (1) sham-operated rats housed in SE (Sham); (2) MCAO rats housed in SE (MCAO); (3) MCAO rats housed in EE (EE); (4) EE rats treated with anti-IL-17A mAb (EE + anti-IL-17A); (5) EE rats treated with mouse IgG1 isotype (EE + IgG1 isotype). In more detail, for group (4), anti-IL-17A mAb (2 μg/rat; Sigma-Aldrich, St Louis, MO, USA) was administered intracerebroventricularly (i.c.v.) into the left lateral ventricle (coordinates:+0.8 mm posteriorly, +1.5 mm lateral from the bregma, –3.5 mm depth) using a stereotaxic instrument (ZH-Lanxing B-type stereotaxic frame, Anhui Zhenghua Biological Equipment Co., Ltd., Huaibei, China) every 24 h for 1 week starting at 14 days post-ischemia (dpi). I.c.v. injection was performed with a sterile 26G Hamilton microsyringe (80330; Hamilton Company, Reno, NV, USA). Moreover, for group (5), 2 μg IgG1 isotype control anti-IL-17A mAb was administered i.c.v. into the left lateral ventricle of EE rats every 24 h for 1 week starting at 14 dpi. A schematic representation of the experimental timeline is presented in Figure 1A.

### Behavioral tests

A set of behavior tests, including the modified neurological severity scores (mNSS) test and rotarod test, were carried out at 3, 7, 14, and 21 days post-MCAO by an investigator who was unaware of the experimental groups (*n* = 12/group).

The mNSS was used to comprehensively assess motor, sensory, balance, and reflex functions according to the method previously reported in Wu et al. (2021). The range of the neurological function score extends from 0 to 18 points (maximal deficit score, 18; normal score, 0) and was recorded at 3, 7, 14, and 21 days after MCAO surgery. A higher score represents more severe neurological deficits.

The rotarod test (LE8200 Panlab, Harvard Apparatus, United States) was conducted to provide an index of forelimb and hindlimb motor coordination and balance. Rats were tested under the accelerating rotor mode with acceleration from 5 to 40 rpm for in 300 s. The length of time that the animal remained on the rod was noted by an investigator who was blinded to the experimental groups. The final score for statistical analysis was expressed as the mean time that a rat could remain on the rod over two trials (Zhang et al., 2021).

### 2,3,5-triphenyl tetrazolium chloride (TTC) staining for infarct volume

Rats (*n* = 6 per group) were narcotized by an intraperitoneal overdose injection of chloral hydrate and sacrificed by decapitation at 21 days after MCAO. Brains were removed carefully to preserve brain integrity. The brains were kept in a –20°C freezer for 20 min prior to being sliced into six coronal sections (2 mm thick) and immersed in a solution of 2% 2,3,5-triphenyl tetrazolium chloride (TTC, Sigma, USA) in phosphate-buffered saline (PBS) for 30 min at 37°C in darkness, then fixed in 4% paraformaldehyde overnight. To minimize the effects of edema and liquefaction resulting from infarction, the percentage of infarct volume was counted according to the method previously reported in (Liao et al., 2020). Infarct volumes were calculated by adding up the infarct areas in six brain slices by thickness (2 mm). The percentage of infarct volume = [(contralateral hemispheric volume–ipsilateral non-infarct volume)/contralateral hemispheric volume] × 100%. The brain slices were photographed and the infarct size was calculated using Image Pro Plus 6.0 (Media Cybernetics Inc., Bethesda, MD, USA) by an observer without knowledge of the experiment.

### Immunofluorescence

Rats (*n* = 6/group) were transcardially perfused with 4% paraformaldehyde, and the penumbra tissues were fixed in 4% paraformaldehyde at 4°C for 24 h, after which they were embedded in paraffin and cut into coronal sections of 3 μm thickness. The primary antibody used for immunoassays was as follows: rat polyclonal glial fibrillary acidic protein (GFAP) (1:200, Abcam, ab279291); rabbit polyclonal IL-17A (1:50, PA5-114345, Invitrogen); or rabbit monoclonal CD34 (1:50, Abcam, ab81289). Primary antibodies were detected with Cy3-conjugated goat anti-rabbit secondary (1:100, Aspen, AS-1109) and FITC-conjugated goat anti-rat secondary (1:50, Proteintech Group, SA00003-11). For quantitative analysis, five non-overlapping fields (200 ×) in the penumbra were photographed under fluorescence microscopy (Eclipse Ci-L, Nikon) and recorded by an observer blind to the experimental groups. The quantification of microvascular density (MVD) from five regions of each individual rat was expressed as numbers/mm^2^.

### Western blotting

The tissue samples of ischemic penumbra (*n* = 3)were harvested 21 days after MCAO surgery. The isolated brain cortices were rinsed with precooled PBS two or three times, cut into small sections, and transferred into a homogenizer after blood was removed. Upon total homogenization, the homogenates were placed for 30 min in an ice bath. The protein concentration was determined using a BCA protein assay kit (Aspen, China). Protein (40 μg/lane) was electrophoresed on polyacrylamide gels (SDS-PAGE) prior to transfer to the polyvinylidene fluoride (PVDF) membranes (Millipore, Massachusetts, USA). Blocking solution was then added to the PVDF membranes for 1 h at room temperature. The blocking solution was subsequently discarded, after which the membranes were incubated at 4°C overnight with a primary antibody. After being washed three times (5 min each) with TBST, membranes were then exposed to the corresponding secondary antibody (HRP-Goat anti-rabbit/mouse antibody, HRP-Rabbit anti-goat/rat antibody, 1:10000) at room temperature for 30 min. The mixed enhanced chemiluminescence (ECL) solution was added to the protein side of the membrane before exposure in a dark room. The AlphaEaseFC software processing system (version 3.2β; Alpha Innotech) was utilized to analyze the optical density value of the target bands. The primary antibodies, STAT3 (ab109085), VEGF (ab46154), CD34 (ab81289), and GAPDH (ab181602), were all purchased from Abcam (Cambridge, UK); the primary antibody IL-17A (PA5-114345) was purchased from Thermofisher (Carlsbad, CA, USA); the IL-6 (DF6087) was purchased from Affinity Biosciences (Cincinnati, USA); finally, the JAK2 (#3230) was purchased from Cell Signaling Technology (Beverly, MA, USA).

### Real-time quantitative PCR (rT-qPCR)

Rats (*n* = 3/group) were decapitated following the completion of behavioral testing, and cortical tissue samples were collected. The gene expression of IL-17A, IL-6, VEGF, JAK2, and STAT3 was measured by RT-qPCR as described in previous study (Liu et al., 2021). The RNA expression levels were analyzed using a StepOneTM Real-Time PCR Instrument System (LifeTechnologies). RT-qPCR was performed using a QuFast SYBR Green PCR Master Mix kit (ELK Biotechnology, EQ001), with three replicate wells for each sample. PCR was performed pre-denaturation at 95°C/60 s, 95°C for 15 s, 58°C for 20 s, and 72°C for 45 s for 40 cycles. The primer sequences used for real-time PCR were as follows:

IL-17A (F): 5′- CTCAGACTACCTCAACCGTTCC -3′;

IL-17A (R): 5′- CACTTCTCAGGCTCCCTCTTC -3′;

IL-6 (F): 5′- TGGAGTTCCGTTTCTACCTGG -3′;

IL-6 (R): 5′- GGTCCTTAGCCACTCCTTCTGT -3′;

VEGF (F): 5′- ATCTTCAAGCCGTCCTGTGTG -3′;

VEGF (R): 5′- AGGTTTGATCCGCATGATCTG -3′;

JAK2 (F): 5′- CTCAAAGAAAGCTGCAGTTCTAT -3′;

JAK2 (R): 5′- TAATCTGGAGTAAACAGGCTGTT -3′;

STAT3 (F): 5′- GAGAACTGAGTGAGCGTGGGT -3′;

STAT3 (R): 5′- CGCTTGTCCAACAACAAACC -3′;

GAPDH (F): 5′- AACAGCAACTCCCATTCTTCC -3′;

GAPDH (R): 5′- TGGTCCAGGGTTTCTTACTCC -3′;

### Statistical analysis

Statistical analysis was performed using the SPSS 20.0 statistical software package. All data are presented as means ± standard deviation (SD). The behavioral tests, including the mNSS and rotarod tests, were compared using a two-way analysis of variance (ANOVA); the other data were analyzed by one-way ANOVA. Values of *P* < 0.05 were considered statistically significant.

## Results

### EE promoted functional recovery and decreased infarct volume during the late phase of stroke

In order to test the neurofunctional effects of EE in MCAO rats, the mNSS and rotarod tests were used to assess exercise capacity among the five groups at 3, 7, 14, and 21 days after MCAO. As shown in Figure 1B, the mNSS scores of the sham group were 0. There was no significant difference between MCAO groups (MCAO, EE, EE + anti-IL-17A, and EE + isotype) on day 3. From day 7 to 21, the mNSS scores decreased gradually in ischemic rats, while the EE, EE + anti-IL-17A, and EE + isotype groups exhibited better functional recovery compared with the MCAO group (*P* < 0.05, Figure 1B). By day 14 and 21, the mNSS scores in the EE + anti-IL-17A group were significantly lower than those in the MCAO group, while a further decrease was observed in the EE and EE + isotype groups (*P* < 0.05, Figure 1B).

In the rotarod test, the average time on the rod was significantly higher in the EE and EE + isotype groups compared with the MCAO group or the EE + anti-IL-17A group at 7 days (*P* < 0.05, Figure 1C), but no significant difference was found between the MCAO and EE + anti-IL-17A groups (*P* > 0.05, Figure 1C). Treatment with EE, isotype, and anti-IL-17A significantly increased the time on the rod compared with the MCAO group at day 14 and 21 (*P* < 0.05, Figure 1C), while rats in the EE and EE + isotype groups exhibited significantly higher time on the rod than those in the EE + anti-IL-17A group (*P* < 0.01, Figure 1C).

For the TTC testing, the infarct volume for each rat was measured 21 days after stroke. As the TTC staining revealed, the infarct volume of the three EE-treated groups (EE group, EE + isotype group, and EE + anti-IL-17A group) was clearly smaller than that of the MCAO group (*P* < 0.05, Figures 1D, E). Moreover, the EE and EE + isotype groups exhibited significantly reduced infarct volume compared with the EE + anti-IL-17A group (*P* < 0.01, Figures 1D, E). These results indicate that EE may promote functional recovery and decrease the infarct volume during the late phase of stroke.

### EE increased the expression of astrocytic IL-17A in the ischemic penumbra during the recovery phase of stroke

**FIGURE 2:**
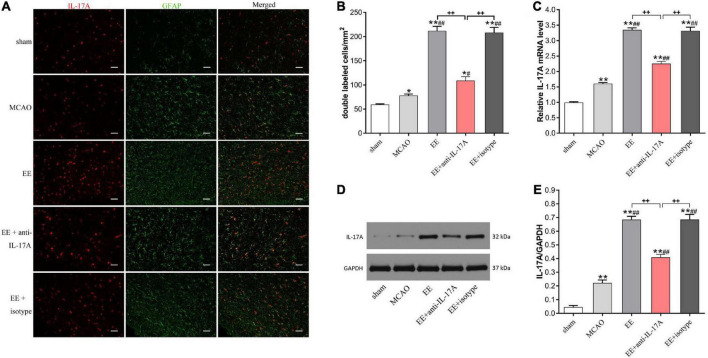
Enriched environment (EE) increased the protein and mRNA expression of interleukin-17A (IL-17A) in astrocytes at 21 days after middle cerebral artery occlusion (MCAO). **(A)** Double immunofluorescence imaging of IL-17A (red) cells co-expressing glial fibrillary acidic protein (GFAP) (green) in penumbra. Bar = 50 μm. **(B)** Quantitative analysis of double-labeled cells in penumbra, *n* = 6. **(C)** Quantitative analysis of IL-17A mRNA levels in penumbra, *n* = 3. **(D,E)** Western blotting and quantification illustrating increasing expression of IL-17A in the ischemic hemisphere, *n* = 3. Data are presented as mean ± SD. *P < *0.05*, **P < *0.01* vs. sham group; ^#^*P* < *0.05*, ^##^*P* < *0.01* vs. MCAO group; ^++^*P* < *0.01* vs. EE + anti-IL-17A group.

To explore whether IL-17A was derived from astrocytes and involved in EE-induced neuroprotection, we studied the protein expression levels of IL-17A in the penumbra 21 days after cerebral I/R injury. Our data revealed that the number of IL-17A and GFAP cells that were double-labeled by immunofluorescence was significantly increased in MCAO rats (*P* < 0.05, Figures 2A, B). Rats in EE groups showed a higher number of double-labeled cells compared with the MCAO group (*P* < 0.05, Figure 2B). However, the promoting effects of EE on IL-17A levels were attenuated in the EE with the anti-IL-17A treatment group (*P* < 0.01, Figure 2B). Consistent with the above results, both our western blotting and PCR data demonstrated that the mRNA and protein levels of IL-17A in all stroke rats compared with the sham group (*P* < 0.05, Figures 2C–E). The expression of IL-17A was also significantly increased in EE-treated groups relative to the standard condition (MCAO group) (*P* < 0.01, Figures 2C–E). Treatment with EE and EE + isotype upregulated IL-17A expression, while EE + anti-IL-17A had the opposite effect (Figures 2C–E). These data demonstrate that EE increased the expression of astrocytic IL-17A at the recovery phase of stroke.

### IL-17A was involved in EE-mediated angiogenesis in the ischemic penumbra after stroke

**FIGURE 3:**
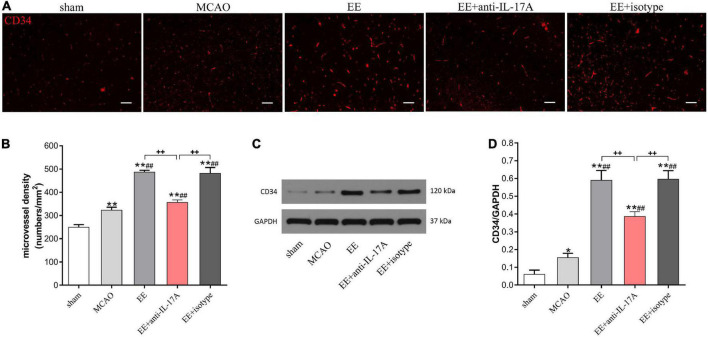
Enriched environment (EE) promoted angiogenesis in penumbra in an interleukin-17A (IL-17A)-dependent manner in penumbra 21 days after middle cerebral artery occlusion (MCAO). **(A)** Representative images of CD34 immunostaining for each group. Bar = 50 μm. **(B)** Quantitative analysis of microvascular density (MVD) in the brain sections in the penumbra of each group, *n* = 3. **(C,D)** Western blotting and quantitative data for CD34 in the ischemic hemisphere, *n* = 3. Data are presented as mean ± SD. *P < *0.05*, **P < *0.01* vs. sham group; ^##^*P* < *0.01* vs. MCAO group; ^++^*P* < *0.01* vs. EE + anti-IL-17A group.

To investigate whether EE-mediated angiogenesis was involved in the promoting effects of astrocytic IL-17A, the MVD in the penumbra was assessed using CD34 by immunofluorescent staining and western blotting 21 days after MCAO. As the results clearly show, EE treatment significantly increased the amount of CD34-positive MVD in the penumbra compared with that in the MCAO group (*P* < 0.01, Figures 3A, B). However, treatment with IL-17A-neutralizing antibodies (EE + anti-IL-17A) neutralized the promoting roles of EE on the MVD (*P* < 0.01, Figures 3A, B). Western blot analysis further showed that MCAO surgery led to a higher expression of CD34 than that in the sham group (*P* < 0.01, Figures 3C, D); however, no difference was found between the EE group and the EE + isotype group (Figures 3C, D). EE rats treated with anti-IL-17A antibodies exhibited significantly reduced protein expression of CD34 in penumbra at 21 days after stroke compared with those in the EE and EE + isotype groups (*P* < 0.01, Figures 3C, D).

### EE promoted VEGF expression after cerebral I/R injury

**FIGURE 4:**
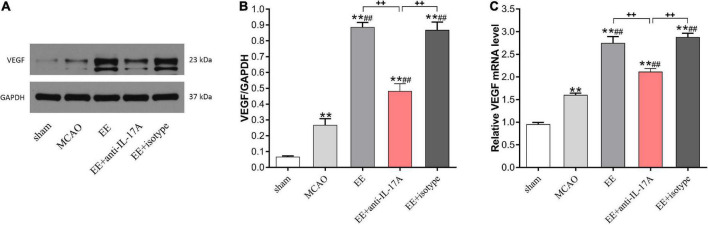
Enriched environment (EE) treatment increased vascular endothelial growth factor (VEGF) protein and mRNA expression in penumbra in an interleukin-17A (IL-17A)-dependent manner 21 days after ischemic stroke. Western blotting **(A)** and quantification analysis **(B)** of VEGF expression in the five groups. **(C)** Quantitative analysis of VEGF mRNA levels in penumbra. Data (*n* = 3/group) are presented as mean ± SD. **P < *0.01* vs. sham group; ^##^*P* < *0.01* vs. MCAO group; ^++^*P* < *0.01* vs. EE + anti-IL-17A group.

To further elucidate the role of IL-17A on EE-mediated angiogenesis, we next evaluated the expression levels of VEGF, a well-studied growth factor for neovascularization, in the penumbra 21 days after stroke. Data from western blotting revealed that the protein levels of VEGF were increased following I/R injury compared with the sham group, and that EE treatment further reinforced this trend (P < 0.01, Figures 4A, B). Rats in the EE and EE + isotype groups showed higher protein levels of VEGF compared with those in the EE + anti-IL-17A group, while no variability was found in these two groups (*P* < 0.01 and *P* > 0.05, Figures 4A, B). The same results were obtained by qRT-PCR. The VEGF mRNA expression levels were significantly increased following MCAO when the rats were housed in an EE cage, while neutralization of IL-17A with anti-IL-17A significantly reversed the promoting effect of EE (*P* < 0.01, Figure 4C).

### Neutralization of IL-17A with anti-IL-17A inhibited IL-6, JAK2, and STAT3 activities in EE-mediated angiogenesis during stroke recovery

**FIGURE 5:**
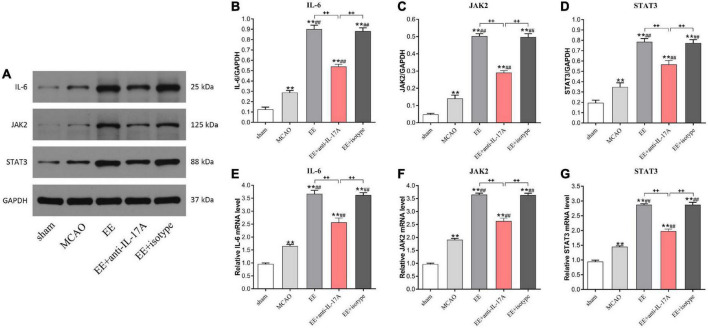
Enriched environment (EE) treatment increased the protein and mRNA levels of the angiogenesis-associated factors IL-6, JAK2, and STAT3 in the penumbra in an interleukin-17A (IL-17A)-dependent manner 21 days after middle cerebral artery occlusion (MCAO). **(A–D)** Western blotting and quantification of angiogenesis-related proteins including IL-6, JAK2, and STAT3 in penumbra. **(E–G)** Quantitative analysis of IL-6, JAK2, and STAT3 mRNA levels in penumbra. Data (*n* = 3/group) are presented as mean ± SD. **P < *0.01* vs. sham group; ^##^*P* < *0.01* vs. MCAO group; ^++^*P* < *0.01* vs. EE + anti-IL-17A group.

To determine whether anti-IL-17A antibodies could suppress EE-mediated angiogenesis in MCAO rats, the protein and mRNA levels of the angiogenesis-related factors IL-6, JAK2, and STAT3 were examined by western blotting and qRT-PCR. As shown in Figure 5, the protein levels of IL-6, JAK2, and STAT3 in the penumbra of MCAO rats were significantly higher than those in sham rats (*P* < 0.01, Figures 5A–D); however, these elevations in EE and EE + isotype groups were remarkedly inhibited by the anti-IL-17A (*P* < 0.01, Figures 5B–D). Coincidence with the protein change in IL-6, JAK2, and STAT3 in the penumbra of the cortex, EE treatment also induced an obvious increase in IL-6, JAK2, and STAT3 mRNA expression compared with the standard conditions (*P* < 0.01, Figures 5E–G). This promoting effect induced by EE was also significantly inhibited by the anti-IL-17A antibodies, suggesting that anti-IL-17A could suppress the expression of angiogenesis-related factors induced by EE treatment.

## Discussion

Our previous studies have illustrated that EE treatment-induced neurofunctional recovery was associated with astrocyte proliferation and angiogenesis in the penumbra following experimental stroke. However, the question of whether astrocytes promote tissue repair and angiogenesis *via* some intermediate factors, such as astrocytic IL-17A, needs further exploration. In this study, we demonstrated that EE promoted neurofunctional improvement, reduced infarct volume, and enhanced angiogenesis in the penumbra cortex in experimental animals and, moreover, that astrocytic IL-17A participated in EE-mediated angiogenesis in MCAO rats during the recovery phase of stroke.

The pathophysiological changes after cerebral I/R injury include not only neuronal necrosis but also astrocyte reactivity. The largest and most numerous glial cells in the central nervous system are astrocytes, which provide structural support, maintain normal metabolic activity for neurons, regulate neurotransmitter synthesis, and defend against oxidative stimulation (Gonçalves et al., 2019). Astrocytes play an important role in neuroplasticity (Yew et al., 2019) and are key constituent parts of the blood-brain barrier, which contributes to maintaining brain homeostasis (Aharoni et al., 2021). Previous studies have shown that inhibiting astrocyte activation can simultaneously inhibit the reconstruction and functional recovery of neurovascular units, suggesting that the activation of astrocytes aids in repairing nerve injury in the later stage of cerebral ischemia (Hayakawa et al., 2010). IL-17A was found to be mainly derived from astrocytes and to augment neurogenesis and functional recovery after stroke (Zhang et al., 2016). Zhang et al. (2016) showed that astrocytic IL-17A promoted angiogenesis in the ischemic penumbra during stroke recovery and further proved that either anti-IL-17A mAb treatment or IL-17A knock-out attenuated the angiogenesis promotion effects. Furthermore, ocular angiogenesis was also found to be promoted by IL-17A expression *in vivo* (Li and Zhou, 2019). *In vitro*, recombinant IL-17A (rIL-17A) may promote corneal fibroblasts in a mouse corneal injury model, while IL-17A neutralization was found to suppress the growth and migration of endothelial cells and the formation of capillary-like structures (Liu et al., 2017). These results provide evidence that IL-17A may promote neurovascular remodeling after ischemic injury. Consistent with these previous studies, our findings highlighted the important role played by EE-induced astrocytic IL-17A expression in the penumbra in promoting functional recovery after cerebral I/R injury, as shown by the evidence that the EE and EE + IgG1 isotype conditions significantly enhanced IL-17A protein levels. In addition, it was also observed that IL-17A promoted angiogenesis in the ischemic penumbra. Anti-IL-17A mAb treatment decreased CD34-positive MVD in the peri-infarct ischemic area. In future studies, we should involve overexpression IL-17A to further determine the relationship between EE-induced angiogenesis and IL-17A. Based on our previous work and the current research, we further concluded that EE-induced astrocytic IL-17A improves neurofunctional recovery *via* promoting angiogenesis after experimental stroke. Less perfection of our study was the lack of IL-17A knockdown out or overexpression experiments to verify the deterministic effect of EE on the expression of IL-17A. Further studies should be conducted on this aspect in the future.

Multiple factors participate in angiogenesis, including astrocytes, vascular endothelial cells, and regulatory factors (Schmidt et al., 2013). The degree and scope of newly generated angiogenesis are closely related to the prognosis for patients with cerebral ischemia. VEGF is the key regulatory factor in the process of angiogenesis, as it binds with endothelial cell surface receptors, initiates cellular cascade, and activates downstream signaling, thereby promoting angiogenesis and function recovery. Previous research has also shown that the VEGF gene plays a vital role in both angiogenesis and neurogenesis (Wright et al., 2019). Zhang et al. (2016) demonstrated that VEGF was involved in IL-17A’s promotion of angiogenesis and functional recovery after stroke, while a neutralizing antibody of IL-17A was found to inhibit VEGF expression and attenuate the promoting effects of rIL-17A on CD34 expression in the penumbra. As a marker of neoangiogenesis, the endothelial antigen CD34 has been used to highlight the microvessel density (Fei et al., 2018). A previous study has demonstrated that higher VEGF expression levels increased the density of microvessels in experimental breast cancer (Huang et al., 2013). Abdel-Razik et al. (2020) also confirmed that an increase in VEGF concentration was associated with a significant increase in neoangiogenesis as measured by CD34-positive microvessels. In this study, our data provide evidence that EE treatment promoted angiogenesis by increasing the protein expression of VEGF and CD34 in the ischemic penumbra through astrocytic IL-17A during the recovery phase after stroke. However, the promoting effects of EE treatment on VEGF and CD34 protein levels were found to be attenuated by anti-IL-17A mAb treatment.

The promoting effect of IL-17A on angiogenesis was associated with the expression of IL-6 during the stroke recovery stage. Previous research has revealed that IL-6 significantly enhanced the formation of angiogenesis and functional recovery (Droho et al., 2021). As a pleiotropic cytokine, IL-6 is an important messenger molecule among leukocytes, endothelial cells, and parenchyma cells (Gertz et al., 2012). IL-6 might play multiple competing roles in anti-apoptosis, growth inhibitory, promoting proliferation and inducing differentiation depending on the cellular settings. IL-6 belongs to the glycoprotein 130 activating cytokine family, which interacts with the cell surface receptor IL-6R, including the ligand binding α subunit (IL-6Rα) and transmembrane glycoprotein β subunit (gp130). The combination of IL-6 and IL-6Rα induces the dimerization of gp130, leading to the activation and phosphorylation of STAT3 (Sulaiman et al., 2019). The proangiogenic effect of IL-6 is closely associated with the early activation of angiogenesis-related gene transcription and STAT3 phosphorylation in the delayed phases after stroke (Gertz et al., 2012). Previous research has revealed that EE has a neuroprotective effect following cerebral injury by promoting angiogenesis *via* astrocytic HMGBI/IL-6 pathways, suggesting the involvement of IL-6 in vascular remodeling and angiogenesis (Chen J. et al., 2017). VEGF is considered one of the key drivers in blood vessels, inducing proliferation and new microvessel formation. Multiple studies have reported that VEGF is the main target gene for STAT3 during hypoxia (Liao et al., 2017; Wu X. et al., 2020). STAT3 can be activated by the phosphorylation of JAK2 (p-JAK2) before being translocated to the nucleus to modulate the expression of downstream target genes in the nucleus (Liu et al., 2014). A growing body of evidence suggests that IL-6 is a positive regulator of JAK2/STAT3 signaling, which leads to angiogenic VEGF expression (Subotički et al., 2021); this was also confirmed in a stroke model of mice (Li et al., 2017). Recently, research has suggested that IL-17A-mediated neovascularization is inextricably linked with the IL-6/STAT3 pathway in animal tumor models (Zou and Restifo, 2010). Similarly, IL-17A strongly stimulates VEGF production and drives neovascular growth in animals *via* the STAT3/GIV signaling pathways in non-small-cell lung cancer (Pan et al., 2015). However, after blocking the expression of STAT3 *in vitro* and *in vivo*, a decrease in IL-17A-induced VEGF expression, along with the proliferation of endothelial cells and neovascular development, was also observed (Pan et al., 2015). It should moreover be noted that the pro-angiogenetic effect of IL-17A may be dependent on the IL-6/STAT3 signaling pathways under EE conditions after cerebral ischemia; this matter should be further explored in the future. In this study, our results showed that astrocytic IL-17A promoting post-stroke angiogenesis in enriched animals might be linked with the upregulation of IL-6, JAK2, and STAT3, and moreover, that this promoting effect was neutralized by anti-IL-17 mAb.

By supporting recovery through multiple avenues, EE emerges as a more well-rounded approach to functional recovery after cerebral I/R injury. Our findings shed new light on mechanisms of EE-induced angiogenesis in stroke recovery and suggest a number of potential implications in development of new therapeutic strategies. This also help create a pre-clinical therapy paradigm that more accurately mimics the clinical setting. Neovascularization and perfusion of the vascular structure in the peri-infarct brain have very vital role in functional improvement in experimental stroke. Similarly, promoting angiogenesis provides a novel therapeutic strategy in clinical management of stroke patients. Therefore, findings in experimental models can be relatively easily translated to the clinical settings. Recently, EE has been applied into a rehabilitation strategy for post-stroke patients which provides preliminary support of the implementation of an EE within a tipical rehabilitation setting (White et al., 2015). The knowledge that stroke patients spend too much time alone and inactive, and arising evidence from animal models of stroke, supports studies to apply EE to human stroke survivors. Given limited resources and therapist time, EE-like environment can be applied in clinical practice that provides physical activity, social interaction and challenging tasks to accelerate recovery after stroke. Also, experimental treatments which target angiogenesis and its related molecules might provide theoretical basis for stroke, provides a potential target for the application of EE translation. However, there are still some limitations in our study. Overexpression or knockdown out IL-17A experiments should be conducted to verify the deterministic effect of EE-induced angiogenesis after cerebral I/R injury in the future.

## Conclusion

Our results revealed that post-stroke EE treatment promoted functional recovery in a pro-angiogenesis manner through astrocytic IL-17A, which might promote CD34 and VEGF expression during the recovery phase of stroke. The beneficial effect of EE-mediated angiogenesis may result from the promotion of IL-6, JAK2, and STAT3 expression. This study provides more theoretical evidence that EE is a promising rehabilitation strategy for stroke in future clinical applications and ultimately facilitates translation into the rehabilitation setting.

## Data availability statement

The raw data supporting the conclusions of this article will be made available by the authors, without undue reservation.

## Ethics statement

All animal procedures were reviewed and approved by the Ethics Committee of the Nanchang University at Nanchang, China (No.202012-92).

## Author contributions

XC: conception and design of the research, obtaining financing, and writing of the manuscript. LL: carring out the tissue sampling. YZ: analysis and interpretation of the data. YL: execution of the research and statistical analysis. All authors critical revision of the manuscript for intellectual content, and read and approved the final draft.
