# Supplementary material for: Enriched environment promotes post-stroke angiogenesis through astrocytic interleukin-17A
Source: Front Behav Neurosci. 2023 Feb 16;17:1053877. doi: 10.3389/fnbeh.2023.1053877 (PMC9979086; doi:10.3389/fnbeh.2023.1053877)
Supplement: Supplementary file 1 [file Table_1.DOC]

https://www.jianguoyun.com/p/DTqSRnwQnK_-ChiZutsEIAA
